# Adhesive Performance Assessment of Universal Adhesives and Universal Adhesive/Composite Cement Combinations

**DOI:** 10.3290/j.jad.b4646953

**Published:** 2023-11-17

**Authors:** Chuliang Tang, Ben Mercelis, Mohammed H. Ahmed, Kumiko Yoshihara, Marleen Peumans, Bart Van Meerbeek

**Affiliations:** a PhD Student, KU Leuven (University of Leuven), Department of Oral Health Sciences, BIOMAT – Biomaterials Research group & UZ Leuven (University Hospitals Leuven), Dentistry, Leuven, Belgium. Experimental design, performed experiment and wrote the manuscript.; b Research Group Manager, KU Leuven (University of Leuven), Department of Oral Health Sciences, BIOMAT – Biomaterials Research group & UZ Leuven (University Hospitals Leuven), Dentistry, Leuven, Belgium. Performed part of the experiments.; c Junior Postdoctoral Researcher, KU Leuven (University of Leuven), Department of Oral Health Sciences, BIOMAT – Biomaterials Research group & UZ Leuven (University Hospitals Leuven), Dentistry, Leuven, Belgium, and Tanta University, Faculty of Dentistry, Department of Dental Biomaterials, Tanta, Egypt. Consulted on and performed statistical evaluation.; d Senior Postdoctoral Researcher, National Institute of Advanced Industrial Science and Technology (AIST), Health and Medical Research Institute, Kagawa, Japan; Okayama University, Graduate School of Medicine, Dentistry and Pharmaceutical Sciences, Department of Pathology & Experimental Medicine, Okayama, Japan. Contributed substantially to discussion.; e Professor, KU Leuven (University of Leuven), Department of Oral Health Sciences, BIOMAT – Biomaterials Research group & UZ Leuven (University Hospitals Leuven), Dentistry, Leuven, Belgium. Contributed substantially to discussion.; f Full Professor, KU Leuven (University of Leuven), Department of Oral Health Sciences, BIOMAT – Biomaterials Research group & UZ Leuven (University Hospitals Leuven), Dentistry, Leuven, Belgium. Idea, proofread the manuscript and contributed substantially to discussion.

**Keywords:** adhesion, bonding, light curing, self-curing, bond strength, TEM

## Abstract

**Purpose:**

To investigate the bonding performance of three universal adhesives (UAs) to dentin and the effect of different curing modes and hydrofluoric-acid (HF) etching of lithium-disilicate glass-ceramic on the adhesive performance of two UA/composite cement (CC) combinations.

**Materials and Methods:**

In the first project part, the immediate and aged (25k and 50k thermocycles) microtensile bond strength (µTBS) of the two light-curing UAs G2-Bond Universal (G2B; GC) and Scotchbond Universal Plus (SBUp; 3M Oral Care), and the self-curing UA Tokuyama Universal Bond II (TUBII; Tokuyama) to flat dentin was measured, when applied in both E&R and SE bonding mode using a split-tooth design (n = 10). The resultant adhesive-dentin interfaces were characterized using TEM. In the second project part, CAD/CAM composite blocks were luted to flat dentin with either Scotchbond Universal Plus/RelyX Universal (SBUp/RxU; 3M Oral Care) or Tokuyama Universal Bond II/Estecem II Plus (TUBII/ECIIp; Tokuyama) using different curing modes (AA mode: auto-curing of both adhesive and cement; AL mode: auto-curing of adhesive and light-curing of cement), upon which their immediate and aged (25k and 50k thermocycles) µTBS was measured. In the third project part, the same UA/CC combinations were luted to CAD/CAM glass-ceramic to measure their immediate and aged (6-month water storage) shear bond strength (SBS).

**Results:**

In E&R bonding mode, the performance of G2B, SBUp and TUBII was not significantly different in terms of µTBS, while G2B and SBUp significantly outperformed TUBII in SE bonding mode. No significant difference in µTBS was found between the SBUp/RxU and TUBII/ECIIp UA/CC combinations, regardless of bonding mode, aging time, or curing mode. The cement-curing mode did not significantly influence µTBS, while a significantly higher µTBS was recorded for the UA/CC combinations applied in E&R bonding mode. HF significantly improved the SBS of the UA/CC combinations to glass-ceramic.

**Conclusion:**

The self-curing adhesive performed better when applied in E&R than in SE bonding mode. The curing mode did not influence the adhesive performance of the composite cements, while an E&R bonding mode rendered more favorable adhesion in a self-curing luting protocol. When bonding to glass-ceramic, the adhesive performance of the UA/CC combinations benefited from HF etching.

Contemporary restorative dentistry is today based on reliable and durable adhesion of the restorative material to the dental hard tissues.^6,27^ Dental adhesives can traditionally be divided into etch-and-rinse (E&R) and self-etch (SE) adhesives.^44^ The former employs a separate phosphoric-acid etchant while the latter makes use of acidic functional monomers, such as today’s most common and effective monomer 10-methacryloyloxydecyl dihydrogen phosphate (10-MDP).^26,44,45^ As the newest generation of adhesives, so-called universal adhesives (UAs) can be applied either in a full E&R bonding mode, a full SE bonding mode, or a selective enamel-etching mode, in fact consisting of a combined E&R-on-enamel/SE-on-dentin bonding mode.^28^

Apart from directly restoring teeth by bonding restorative composites to the remaining tooth structure, indirect ceramic/composite restorations are more frequently used in clinical practice to restore larger defective teeth. They offer better control of restoration morphology and function,^25,38^ as well as more favorable optical properties, higher biocompatibility, and longer-term stability.^20^ They also have the advantage of lower-cost, faster and easier fabrication using CAD/CAM techniques,^24,32^ and clinically enable more controlled placement routines. Adhesive luting involves using composite cements that are assisted by E&R or SE adhesives to bond to dentin and enamel.

Although most adhesives are solely light curing and composite cements are mostly dual-curing, self-curing adhesives and luting composites that contain chemical polymerization initiators and thus do not require separate light curing can be an option, especially when adequate light irradiation can hardly be achieved in cases of thicker and opaquer restorations.^15,42^ Nevertheless, dual-cure composite cements have repeatedly been reported to benefit from light curing, revealing better bonding performance than when they are solely left to auto-cure.^4,11,14,20^ For adhesive-assisted composite cements, scientists and manufacturers disagree on whether the adhesive should be separately light-cured prior to the application of the composite cement. Light curing has been claimed to be immediately/separately (prior to composite-cement application) necessary to avoid water uptake from dentin due to osmosis (given vital teeth).^20,21^ Separate and immediate light curing enables the adhesive to polymerize sufficiently (despite some polymerization inhibition by air), importantly hereby stabilizing the adhesive interface. In contrast, if light curing is delayed until after the restoration is seated, light transmission through the restoration is attenuated. Others claim that the adhesive cannot be separately cured, as it may hinder proper fit of the restoration in cases where the adhesive-film thickness already occupies a significant part of the foreseen cement space.^4,5^ However, sufficient air thinning, also avoiding pooling, can reduce the adhesive-film thickness so that sufficient space remains for the luting composite, considering also that cement spaces are typically at least 50 µm deep and most often even larger in the case of CAD/CAM restorations. A cement space up to 120 µm is still considered clinically acceptable.^7^ In addition, the film thickness of (1-step) UAs is commonly less than 10 µm, so that separate light curing will definitely not harm the restoration seating.

When luting semi-direct/indirect CAD-CAM restorations in glass-ceramic, the restoration is conventionally first etched with hydrofluoric acid (HF) to create microscopically retentive sites that result in increased surface area and roughness.^10,23,29,39^ HF etching is followed by silanization to additionally chemically bind to glass-ceramic.^54^ Many of today’s UAs contain silane to simplify the adhesive luting procedure; manufacturers claim that a separate dedicated ceramic primer is no longer needed.^44^ Although studies found that silane incorporated in UAs was not stable in an acidic aqueous solution and hence a separate silane primer was still required,^9,48,49^ new silane technology in the form of γ-methacryloxypropyltriethoxysilane (γ-MPTES) added to a specific UA was reported to be more effective than the previously most commonly used silane coupling agent γ-methacryloxypropyltrimethoxysilane (γ-MPTS).^46^

In this study, the bonding efficacy and durability of two light-curing and one self-curing UA were studied in Project Part 1a using a microtensile bond-strength testing approach, combined with mechanistic TEM characterization of the resultant adhesive-dentin interfaces in Project Part 1b. In Project Part 2, the adhesive luting potential of two universal adhesive/composite cement (UA/CC) combinations employed following two curing regimes was investigated. In Project Part 3, the adhesive luting efficacy to glass-ceramic of the two UA/CC combinations, in which both UAs contained the silane coupling agent γ-MPTES, was evaluated. The effect of HF etching of lithium-disilicate glass-ceramic on the adhesive luting performance of the UA/CC combinations was explored. Project parts 2 and 3 aimed to investigate the additional indication of UAs when combined with CC for adhesive luting purposes of semi-direct/indirect ceramic/composite CAD/CAM restorations. For Project Part 1, the null hypothesis tested was that the bonding efficiency of the self-curing UA would not underperform that of the light-curing adhesives; for Project Part 2, the null hypothesis was that the two UA/CC combinations would not differ in bonding performance and the curing regimes would not affect the luting efficacy of the composite cements; and for Project Part 3, the null hypothesis was that the adhesive luting efficacy of the UA/CC combinations to lithium-disilicate glass-ceramic would not be affected when the glass-ceramic was not etched with HF.

## Materials and Methods

### Bonding Efficiency/Durability of UAs to Flat Dentin (Project Part 1a)

Based on the informed consent approved by the Commission of Medical Ethics of KU Leuven with file number S64350, thirty non-carious human third molars (n = 10/experimental group) were collected and stored in distilled water at 4°C for at least 1 week and up to 1 month.

The bonding efficacy/durability of the two-component self-curing 1-step UA Tokuyama Universal Bond II (TUBII, Tokuyama; Tokyo, Japan), light-curing 1-step UA Scotchbond Universal Plus (SBUp, 3M Oral Care; Seefeld, Germany), and light-curing 2-step UA G2-Bond Universal (G2B, GC; Tokyo, Japan) was investigated.

The collected teeth were randomly allocated into three experimental groups with 10 teeth per group. After cutting the occlusal third of tooth crowns at the level of mid-coronal dentin using a slow-speed diamond saw (Micracut 151, Metkon; Bursa, Turkey), a shallow 300-µm groove was cut at the center of the exposed dentin surface to divide the surface into two equal parts. A standardized smear layer was next prepared using a high-speed medium-grit diamond bur (882.314.014, Komet; Lemgo, Germany), mounted in the MicroSpecimen Former (University of Iowa; Iowa City, IA, USA). Any tooth with remaining enamel or exposure of pulp tissue was excluded upon checking the exposed dentin surface using a stereomicroscope (Stemi 2000-CS, Zeiss; Oberkochen, Germany). Before having applied the dental adhesives, the teeth were warmed in 100% humidity at 37°C for at least 30 min. The split-tooth design was employed by placing a thin razor blade into the groove to separate the dentin surface into halves. Half of the dentin surface was etched with phosphoric acid (Tokuyama Etching Gel HV, Tokuyama) for 15 s and rinsed with water for 15 s; this was the E&R half. The other half was not treated with phosphoric acid and served as the SE half. Upon removal of the blade, the whole dentin surface was rinsed and gently dried. The adhesives investigated were next applied on the exposed dentin surface according to the respective manufacturer’s instructions (Table 1). Next, the restorative resin composite Estelite Asteria (Tokuyama) was applied in 3 layers up to a height of 5 mm, each layer was cured for 20 s, followed by additional light curing for 20 s from each lateral side and the top side. All light curing was done using the LED light-curing unit SmartLite Pro (Dentsply Sirona; Konstanz, Germany), having a light output of at least 1200 mW/cm^2^, as confirmed regularly using a Marc Resin Calibrator (BlueLight Analytics; Halifax, Canada). The specimens were next immediately stored in 100% humidity at 37°C for 24 h, before being transferred to pre-warmed 37°C distilled water for 6 days.

**Table 1:** Composition of the universal adhesives (UAs) and composite cements (CCs) investigated in this study

Material	Composition1	Instructions
G2-Bond Universal [G2B] (GC)	Primer: 10-MDP, 4-MET, dimethacrylate resins, photo-initiator, BHT, water, acetone Bond: UDMA, dimethacrylate, photo-initiator, silica, BHT	E&R: Etch with etching gel HV (Tokuyama) for 15 s, rinse for 15 s, and then proceed as for SE. SE: Apply Primer using a microbrush, leave undisturbed for 10 s, dry thoroughly for 5 s with maximum air pressure, apply bond, gently dry for 5 s and ligh cure for 10 s.
Scotchbond Universal Plus [SBUp] (3M Oral Care)	10-MDP, HEMA, dimethacrylate resins, silanes (APTES/γ-MPTES), silica, ethanol, water, CQ	E&R: Etch with etching gel HV (Tokuyama) for 15 s, rinse for 15 s, and then proceed as for SE. SE: Apply the adhesive and rub for 20 s, air-blow gently for a minimum of 5 s until it does not move anymore, and light cure for 10 s (no light curing when applied with RelyX Universal). Bonding to glass-ceramic: Apply the adhesive and rub for 20 s, air blow gently for a minimum of 5 s until it does not move anymore (no light curing), apply the cement RelyX Universal and light cure for 20 s.
Tokuyama Universal Bond II [TUBII] (Tokuyama)	Etching gel HV: phosphoric acid (39 wt%) Bond A: phosphoric acid monomer (3D-SR monomer), 10-MDP, HEMA, bis-GMA, TEG-DMA, MTU-6, BHT, acetone Bond B: γ-MPTES, borate, peroxide, BHT, acetone, ethanol, water	E&R: Etch with Etching gel HV for 15 s, rinse for 15 s, and then proceed as for SE. SE: Dispense one drop of Bond A and B into a disposable mixing well, apply the mixed bond and immediately gently dry (no light curing). Bonding to glass-ceramic: Dispense one drop of Bond A and B into a disposable mixing well, apply the mixed bond, immediately gently dry (no light curing), apply the cement Estecem II Plus and light-cure for 20 s.
RelyX Universal [RxU] (3M Oral Care)	TEG-DMA, HEMA, ytterbium fluoride, glass powder, silane, silica, titanium dioxide, CQ, peroxide, BHT	Squeeze out a small quantity of Paste A and B, mount the mixing tip and apply the cement on the restoration. AA curing mode: keep in the dark for 6 min. AL curing mode: light cure the cement for 10 s from each lateral side and the top side (50 s in total) in Project Part 2 or for 20 s in Project Part 3.
Estecem II Plus [ECIIp] (Tokuyama)	Bis-GMA, TEG-DMA, bis-MPEPP, silica-zirconia filler, CQ, benzoyl peroxide, silicon dioxide, titanium dioxide, BHT	Squeeze out a small quantity of Paste A and B, mount the mixing tip and apply the cement on the restoration. AA curing mode: keep in the dark for 8 min. AL curing mode: light cure the cement for 10 s from each lateral side and the top side (50 s in total) in Project Part 2 or for 20 s in Project Part 3.
Porcelain Etch (Ultradent)	9% hydrofluoric acid	Apply the etchant to the ceramic surface for 30 s, rinse under a constant stream of tap water for 1 min and dry thoroughly with a clean, oil-free air stream.

1 3D-SR: three dimensional self-reinforcing; 4-MET: 4-methacryloxyethyl trimellitic acid; 10-MDP: methacryloyloxydecyl dihydrogen phosphate; APTES: (3-aminopropyl)triethoxysilane; BHT: butylated hydroxytoluene; bis-GMA: bisphenol A-glycidyl methacrylate; bis-MPEPP: 2,2-bis[(4-methacryloxy polyethoxy) phenyl] propane; CQ: camphorquinone; HEMA: 2-hydroxyethyl methacrylate; MTU-6: 6-methacryloyloxyhexyl 2-thiouracil-5-carboxylate; TEG-DMA: triethylene glycol dimethacrylate; UDMA: urethane dimethacrylate; γ-MPTES: γ-methacryloxypropyl triethoxy silane.

Before the actual test, each tooth was sectioned using a diamond saw (Accutom-50, Struers; Ballerup, Denmark) to obtain micro(μ)-specimens with a base area of 1x1 mm and a height of 8–10 mm. The central six μ-specimens from each half were checked using a stereomicroscope to ensure that the interface did not include enamel. One-third of the μ-specimens were subjected to μTBS testing immediately without thermocycling (0kTC), one-third of μ-specimens were tested after aging by 25,000 thermocycles (25kTC), while the remaining μ-specimens were tested after undergoing aging by 50,000 thermocycles (50kTC). After measuring the dimensions with a digital caliper (Holex, Hoffmann Group; Munich, Germany), each μ-specimen was glued with cyanoacrylate glue (Model Repair II Blue Dentsply-Sankin; Ohtawara, Japan) to BIOMAT jigs and stressed using an LRX testing device (Lloyd; Hampshire, UK) equipped with a load cell of 100 N and a crosshead speed of 1 mm/min until failure to record the force in N. All μ-specimens that failed before testing were regarded as pre-test failures (ptfs) and were included in the mean calculation as 0 MPa. The μTBS-testing protocol strictly followed the *Academy of Dental Materials* guidelines.^3^

The fractured halves of each μ-specimen were collected after μTBS testing, upon which the failure mode was determined using a stereomicroscope as one of four different types: adhesive interfacial failure, cohesive failure in dentin, cohesive failure in composite, and mixed failure (when multiple failure patterns were observed on the failed surface without any single failure pattern accounting for more than 85% of the whole area). Following conventional processing of SEM specimens, including fixation (2.5% glutaraldehyde in 0.1 M sodium cacodylate buffer), gradual dehydration in ethanol (25%, 50%, 75%, 95%, 100%), and drying with hexamethyldisilazane (HMDS), specimens representing each experimental group were selected and gold-sputter coated (JFC-1300, Jeol; Tokyo, Japan) before being examined using scanning electron microscopy (SEM, JSM-6610LV, Jeol).

### Transmission Electron Microscopic (TEM) Adhesive-Dentin Interfacial Characterization (Project Part 1b)

Flat dentin surfaces, originating from two teeth per UA, were prepared as detailed above except for the smear layer, which was produced in a standardized way by wet-polishing using P600-grit SiC-paper (WS Flex 18C, Hermes Schleifmittel; Hamburg, Germany) in a grinding/polishing machine (Buehler Beta Grinder-polisher, Buehler; Lake Bluff, IL, USA). The UAs G2B, SBUp and TUBII were again applied to the exposed dentin surface in E&R and SE bonding mode according to the respective manufacturer’s instructions (Table 1). One layer of the adhesive resin Clearfil SE Bond 2 (Kuraray Noritake; Tokyo, Japan) approximately 1 mm thick was applied on top of the UA and light cured for 20 s. The teeth were next stored in distilled water at 37°C for 1 week before being sectioned into 0.6- to 0.8-mm-thick slabs. Non-demineralized and demineralized specimens were processed for TEM according to a procedure described in detail elsewhere,^43^ including 38-h immersion in acidic Gooding & Stewart fluid (Prosan; Gent, Belgium) for demineralized specimens, fixation in 2.5% glutaraldehyde in 0.1 M sodium cacodylate for at least 12 h, rinsing in 0.1 M sodium cacodylate buffer for 1 min with 3 changes, dehydration in ascending grades of ethanol (25%, 50%, 75%, 95%, 100%) 2 times each and 10 min per treatment, immersion in 99% propylene oxide 3 times with 10 min per treatment, and embedding in epoxy embedding medium (Sigma-Aldrich; St Louis, MO, USA). Ultra-thin sections of 70-90 nm, originating from at least 2 slabs and 2 teeth, were prepared using an ultramicrotome (Ultracut UCT, Leica; Vienna, Austria) equipped with a 45-degree diamond knife (Diatome; Nidau, Switzerland). These were then examined with TEM (JEM-1400 Flash, Jeol), unstained (for non-demineralized sections) or positively stained (for demineralized sections) with UranyLess (Electron Microscopy Sciences; Hatfield, PA, USA) for 8 min and lead citrate (Electron Microscopy Sciences) for 3 min.

### Adhesive Luting Efficacy of UA/CC Combinations to Flat Dentin (Project Part 2)

This project part investigated the adhesive luting efficacy of the two-component self-curing 1-step UA TUBII (Tokuyama) in combination with the dual-curing CC Estecem II Plus (ECIIp; Tokuyama) and the light-curing 1-step UA SBUp (3M Oral Care) in combination with the dual-curing CC RelyX Universal (RxU; 3M Oral Care). Anticipating a worse-case scenario considering the abovementioned light-curing benefits for CC and the above-discussed debate on the need for separate light curing of the adhesive prior to luting, the UA/CC combinations were applied on dentin following not only an AA curing mode, indicating that both UA and CC were allowed to auto-cure and were not additionally light cured, but also an AL curing mode, indicating that UA was allowed to auto-cure while CC was additionally light cured upon luting the composite block.

Forty non-carious human third molars (n = 10/experimental group) were collected and prepared as detailed above, except for the smear layer that was produced in a standardized way by wet-polishing using P600-grit SiC-paper in the grinding/polishing machine. P600-grit SiC paper corresponds to the use of a red-banded (46-grit) diamond, typically used clinically as the final bur when finishing tooth preparation for semi-direct/indirect tooth restorations.^3^

CAD-CAM composite blocks (Estelite Block II, Tokuyama) were sectioned using the high-speed diamond saw to produce 8x8x5 mm blocks. The block surface to be bonded was additionally ground using P600-grit SiC-paper, followed by sandblasting (Cojet Prep, 3M Oral Care) using Cojet Sand (30-µm silica-coated aluminum oxide sand; 3M Oral Care) with 0.2-MPa pressure for 15 s while keeping a 1-cm distance, and then cleaned in distilled water for 10 min using an ultrasonic bath (Branson 5800, Branson; Danbury, CT, USA). Before luting, the composite-block surface to be luted was coated with the respective UA without light curing, while kept shielded from light. This procedure was recommended by the application instructions of both UAs.

The dental adhesive was next applied on the exposed dentin surface according to the manufacturer’s instructions (Table 1), without separate light curing following both the AA and AL curing mode. Automix composite cement was deposited on the pretreated composite block, upon which the block was positioned on dentin; once seated, it was loaded with 1 kg for 1 min using a BIOMAT custom-made loading device to ensure the load was applied at the block’s center. Only a small but sufficient amount of CC was applied to limit cement excess, which did not require removal of cement excess. Immediately upon loading, glycerine air-block gel (Aquasonic 100, Parker Laboratories; Fairfield, CT, USA) was applied to the cement to reduce polymerization inhibition by oxygen. After luting, the specimens were kept in the dark following the AA curing mode (8 min for TUBII/ECIIp and 6 min for SBUp/RxU, as per setting time indicated by the respective manufacturer), or – following the AL curing mode – light cured for 10 s from each lateral aspect as well as the top (50 s in total) using the LED light-curing unit SmartLite Pro (Dentsply Sirona). After removing the air-block gel, the specimens were stored in 100% humidity at 37°C for 24 h, before being placed in pre-warmed 37°C water for 6 days. The specimens were subsequently sectioned and tested using the same μTBS testing protocol, as detailed above.

The fractured halves of each μ-specimen were collected after μTBS testing, and the failure mode was determined as one of 6 types using the stereomicroscope: cohesive failure in dentin, adhesive failure at the cement-dentin interface, cohesive failure in cement, adhesive failure at the cement-composite interface, cohesive failure in composite, and mixed failure. Representative SEM specimens from all experimental groups were prepared and examined following the same protocol as described above.

### Adhesive Luting Efficiency of UA/CC Combinations to Lithium-Disilicate Glass-Ceramic (Project Part 3)

This project part investigated the adhesive luting efficacy of the same two UA/CC combinations (TUBII/ECIIp, SBUp/RxU) to IPS e.max CAD (Ivoclar; Schaan, Liechtenstein) blocks when treated with or without hydrofluoric acid (HF).

Cross-sectioned glass-ceramic blocks, embedded in acrylic resin, were ground for 1 min in the grinding/polishing machine using P600-grit SiC paper. All surfaces were carefully verified for homogeneous surface treatment, upon which they were randomly divided into 8 experimental groups (n = 10/experimental group). Prior to the adhesive luting procedures, the IPS e.max CAD (Ivoclar) blocks were warmed to 37°C in an incubator at 100% humidity for at least 30 min. A hole with a 3-mm diameter was punched in a piece of double-sided tape prior to sticking the tape to the prepared glass-ceramic surface, with the hole centered in respect to the exposed area. The glass-ceramic blocks were either left without further pre-treatment (NT: no treatment) or were etched with Porcelain Etch (HF: hydro-fluoric-acid etchant, Ultradent; South Jordan, UT, USA) for 30 s. For the HF groups, HF-etching gel was applied on the surface delimited by the hole in the tape for 30 s, after which the sample was thoroughly rinsed under a stream of tap water for 1 min. The specimens were next dried with clean and oil-free air.

The respective UA was then applied on the etched/non-etched glass-ceramic surface according to the manufacturer’s instructions (Table 1). The UAs were not separately light cured. A small portion of automix cement was then applied to the specimen area delimited by the hole in the tape using the automix syringe; SBS testing was performed in a standardized Ultradent jig (Ultradent). Upon placement, the cement (along with the UA) was light cured for 20 s using the LED light-curing unit SmartLite Pro (Dentsply Sirona). Cement excess was carefully removed after light curing using a razor blade.

After preparation, all specimens were stored in 100% humidity at 37°C for 1 h, after which the specimens were transferred into pre-warmed water (37°C) for 1 week. After 1 week, all immediate specimens were subjected to a shear bond-strength (SBS) test using a universal testing machine (Instron 5848 MicroTester; Norwood, MA, USA), when applying a crosshead speed of 1 mm/min until failure occurred. For the actual SBS test, the specimens were positioned in the Ultradent specimen holder according to the ISO 29022 standard.

All fractured specimens were collected and observed using the stereomicroscope to determine the failure modes (one of four types): cohesive failure in glass-ceramic, adhesive failure at the cement-ceramic interface, cohesive failure in cement, and mixed failure.

### Statistical Analysis

Two different linear mixed-effects models (LME; R-4.0.3; R Foundation for Statistical Computing, Vienna, Austria) were built to statistically analyze the outcome of the two μTBS studies at a significance level of α = 0.05:^31^ LME model A (LME-A) in Project Part 1a (μTBS of UAs), involving the three variables ‘adhesive’, ‘aging’ and ‘bonding mode’ as fixed factors, and the variable ‘tooth’ as a random factor; LME model B (LME-B) for Project Part 2 (μTBS of UA/CC combinations), involving the four variables ‘adhesive’, ‘aging’, ‘bonding mode’ and ‘curing’ as fixed factors, and the variable ‘tooth’ as a random factor. For Project Part 3, the SBS data were statistically analyzed using the Kruskal-Wallis test followed by post-hoc Dunn’s test with Bonferroni correction and the Mann-Whitney U-test at a significance level of α=0.05.

## Results

### Bonding Efficacy/Durability of UAs to Flat Dentin (Project Part 1a)

**Fig 1:**
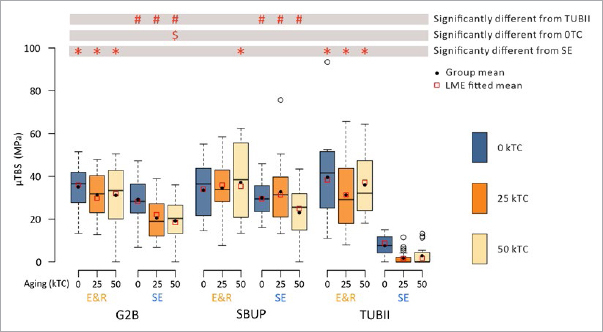
Box-and-whisker plots (Project Part 1a) of the 0kTC, 25kTC-aged and 50kTC-aged μTBS of the universal adhesives (UAs) G2B, SBUp and TUBII to flat dentin when applied in E&R and SE bonding mode. The thick horizontal line within each box represents the median μTBS. The black dot within each box represents the mean μTBS. The red square within each box represents the LME fitted mean. The horizontal lines in each box represent, from top to bottom, the maximum μTBS, the upper quartile, the median μTBS, the lower quartile and the minimum μTBS measured for each experimental group (excluding possible outliers). Statistically significant differences in μTBS with the respective reference are indicated using distinct characters.

All μTBS data are numerically detailed in Table 2 and graphically presented as boxplots in Fig 1. As indicated in Table 3, all three variables significantly contributed to the statistical model. For the interactions, only the adhesive x bonding mode interaction was significant, meaning that the bonding mode had a different impact on the μTBS of the different adhesives, and vice versa.

**Table 2:** Microtensile bond strength (µTBS) of the universal adhesives (UAs) to flat dentin

µTBS (MPa)*	E&R			SE		
	0kTC	25kTC	50kTC	0kTC	25kTC	50kTC
G2B	35.1 ± 10.1 (0/20)	31.3 ± 11.2 (0/20)	31.2 ± 14.0 (1/20)	29.2 ± 10.2 (0/20)	20.5 ± 10.0 (0/20)	19.1 ± 10.2 (2/20)
SBUp	33.6 ± 12.8 (0/20)	34.4 ± 12.6 (0/20)	37.2 ± 17.1 (0/20)	30.0 ± 8.6 (0/20)	32.8 ± 14.9 (0/20)	23.1 ± 11.7 (2/20)
TUBII	39.5 ± 18.4 (0/20)	31.3 ± 16.6 (0/20)	36.0 ± 13.8 (0/19)	7.5 ± 5.1 (4/20)	1.7 ± 3.3 (15/20)	2.9 ± 4.4 (12/20)

* Mean ± SD (ptf/n); SD: standard deviation; ptf: pre-test failure; n: number of µ-specimens.

**Table 3:** Statistical analysis of the fixed variables and interactions of LME model A

	numDF	denDF	F-value	p-value
(Intercept)	1	318	696.7084	<.0001*
Adhesive	2	27	12.7238	0.0001*
Bonding mode	1	318	175.6980	<.0001*
Aging	2	318	5.2109	0.0059*
Adhesive x bonding mode	2	318	43.5575	<.0001*
Adhesive x aging	4	318	2.1526	0.0742
Bonding mode x aging	2	318	2.6891	0.0695

*Statistically significant.

No difference in μTBS was recorded when the three UAs were applied in E&R bonding mode. When applied in SE bonding mode, the μTBS of TUBII was significantly lower than that of G2B and SBUp, this for all three 0kTC (control), 25kTC-aged or 50kTC-aged groups. A significant decrease in μTBS upon aging was only recorded for G2B when applied in SE bonding mode and after 50kTC aging. Comparing the E&R versus SE bonding modes, the μTBS of G2B and TUBII was significantly higher when applied in E&R than in SE bonding mode, regardless of the aging period. For SBUp, only upon 50k TC aging, the μTBS was significantly higher when applied in E&R than in SE bonding mode.

**Fig 2:**
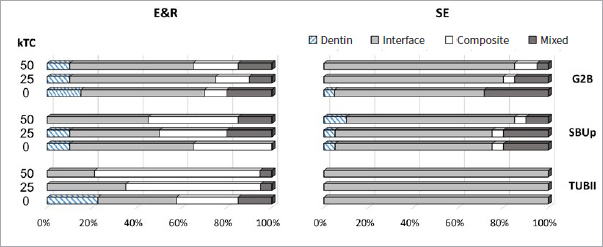
Failure-mode distribution (Project Part 1a) of the G2B, SBUp and TUBII μ-specimens upon μTBS testing when bonded to flat dentin in E&R and SE bonding mode. Dentin: cohesive failure in dentin; Interface: adhesive interfacial failure; composite: cohesive failure in composite; mixed: mixed failure.

The failure mode distribution examined by stereomicroscopy for the adhesives bonded to flat dentin is shown in Fig 2. The failure patterns of G2B, SBUp and TUBII applied in E&R bonding mode to dentin were relatively similar, except that the majority of G2B and SBUp failures were categorized as adhesive interfacial failure, and for TUBII as cohesive failure in composite (except for 0kTC). A less balanced failure mode distribution was recorded when the three adhesives were applied in SE bonding mode, as the predominant failure pattern was adhesive interfacial failure, this even 100% for TUBII.

**Fig 3:**
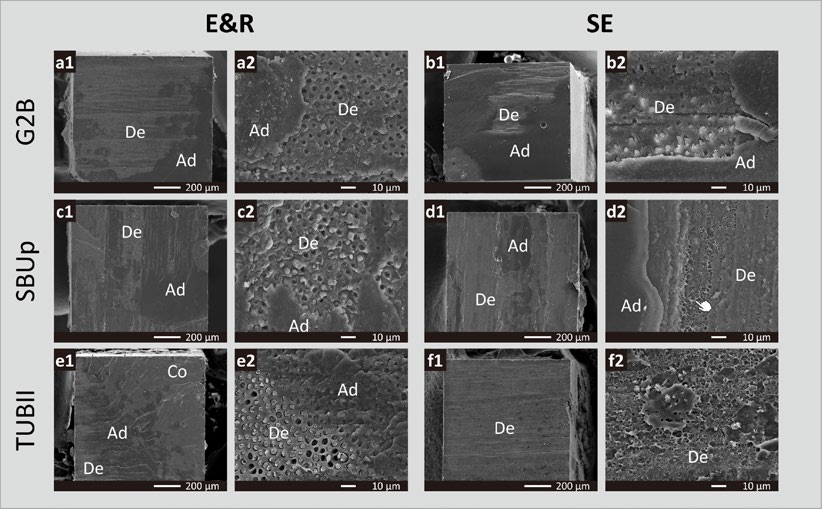
Representative SEM photomicrographs (Project Part 1a) of μ-specimens (dentin side) illustrating the failure mode (1) when G2B was bonded to flat dentin in E&R bonding mode in (a1), revealing a mixed failure enlarged in (a2), and when applied in SE mode in (b1), revealing a mixed failure enlarged in (b2), (2) when SBUp was bonded to flat dentin in E&R bonding mode in (c1), revealing a mixed failure enlarged in (c2), and when applied in SE mode in (d1), revealing a mixed failure enlarged in (d2) and some interfacial voids (hand pointer), and (3) when TUBII was bonded to flat dentin in E&R bonding mode in (e1), revealing a mixed failure enlarged in (e2), and when applied in SE mode in (f1), revealing voids nearly across the whole surface as enlarged in (f2). Ad: adhesive resin; Co: composite; De: dentin.

Representative SEM photomicrographs of fractured μ-specimens (dentin side) are presented in Fig 3, mostly illustrating mixed failure modes. While few interfacial voids were observed along the failed dentin surface when SBUp was applied in SE Bonding mode, the failed surface representing TUBII applied in SE bonding mode disclosed interfacial voids along the entire fractured interface.

### TEM Adhesive-Dentin Interfacial Characterization (Project Part 1b)

**Fig 4:**
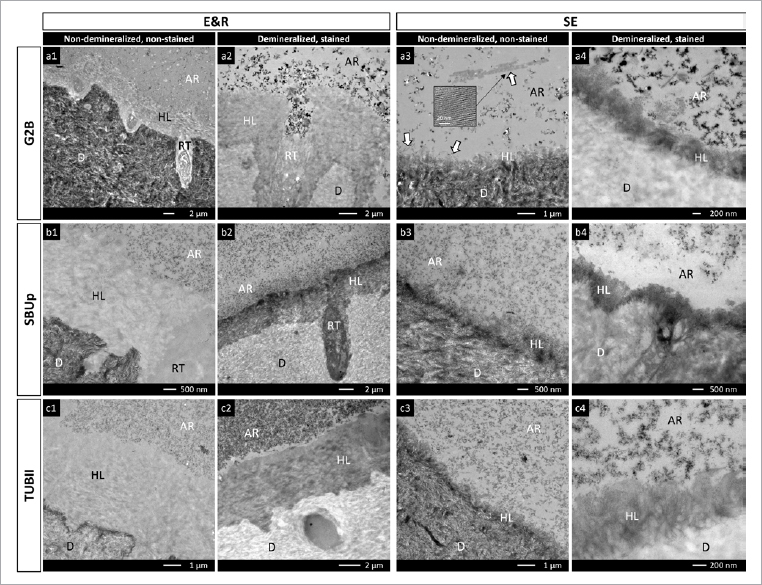
Representative TEM photomicrographs (Project Part 1b) illustrating the ultra-structure of the adhesive-dentin interface produced by G2B in (a1-a4), SBUp in (b1-b4) and TUBII in (c1-c4), when bonded to flat dentin following an E&R and SE bonding mode. a1: non-demineralized, non-stained section of the adhesive-dentin interface produced by G2B applied in E&R bonding mode, revealing a tight bond consisting of a hybrid layer of about 4-5 µm with an abrupt transition to the underlying unaffected dentin. a2: demineralized, stained section of the adhesive-dentin interface produced by G2B applied in E&R mode, revealing a particle-filled resin tag surrounded by an hybridized tubule wall within an opened dentinal tubule. a3: non-demineralized, non-stained section of the adhesive-dentin interface produced by G2B applied in SE mode, disclosing a thin HAp-rich hybrid layer of about 0.5 µm with a gradual transition to unaffected dentin. Significant nano-layering was detected (white arrows) in the adhesive resin above the interface, as magnified in the black-bordered insert. a4: demineralized, stained section of the adhesive-dentin interface produced by G2B applied in SE mode, revealing a clearly defined hybrid layer of about 0.5 µm. b1: non-demineralized, non-stained section of the adhesive-dentin interface produced by SBUp applied in E&R mode, revealing a tight bond consisting of an HAp-free, collagen-rich hybrid layer of about 3-4 µm with an abrupt transition to the underlying unaffected dentin. b2: demineralized, stained section of the adhesive-dentin interface produced by SBUp applied in E&R mode, revealing a well-defined hybrid layer with a resin tag formed within an opened dentinal tubule. b3: non-demineralized, non-stained section of the adhesive-dentin interface produced by SBUp applied in SE mode, disclosing a partially demineralized hybrid layer of 0.4-0.8 µm with a gradual transition to unaffected dentin. b4: demineralized, stained section of the adhesive-dentin interface produced by SBUp applied in SE mode, revealing a distinct 0.6-µm hybrid layer. c1: non-demineralized, non-stained section of the adhesive-dentin interface produced by TUBII applied in E&R mode, revealing a completely demineralized hybrid layer of around 5.0 µm with an abrupt transition to the underlying unaffected dentin. c2: demineralized, stained section of the adhesive-dentin interface produced by TUBII applied in E&R mode, showing an homogeneously stained hybrid layer and densely silica-filled adhesive resin. c3: non-demineralized, non-stained section of the adhesive-dentin interface produced by TUBII applied in SE mode, disclosing a thin, HAp-rich hybrid layer of about 0.4 µm with a gradual transition to unaffected dentin. c4: demineralized, stained section of the adhesive-dentin interface produced by TUBII applied in SE mode, revealing a distinct and homogeneously stained hybrid layer. AR: adhesive resin; D: dentin; HL: hybrid layer; RT: resin tag.

Representative TEM photomicrographs of the adhesive-dentin interfacial ultra-structure of G2B, SBUp and TUBII bonded to flat dentin are illustrated in Fig 4. All adhesives revealed tightly bonded interfaces without interfacial debonding during preparation and imaging of the specimens/sections.

A thicker (3-5 μm) hybrid layer was produced by the UAs applied in E&R bonding mode than when they were applied in SE bonding mode (0.3-0.8 μm). The E&R hybrid layer of the UAs was typically HAp-free with an abrupt transition to the underlying unaffected dentin. Collagen fibrils, sporadically exhibiting cross-banding, could be observed within the E&R hybrid layers on images of stained, demineralized sections. The SE hybrid layer produced by the UAs was only partially demineralized, with abundant HAp still surrounding collagen fibrils within the submicron hybrid layer. The transition to unaffected dentin was more gradual. Overall, no major differences in interfacial ultra-structure were observed by TEM for the three UAs investigated when applied either in E&R or SE bonding mode.

### Adhesive Luting Efficacy of UA/CC Combinations to Flat Dentin (Project Part 2)

**Fig 5:**
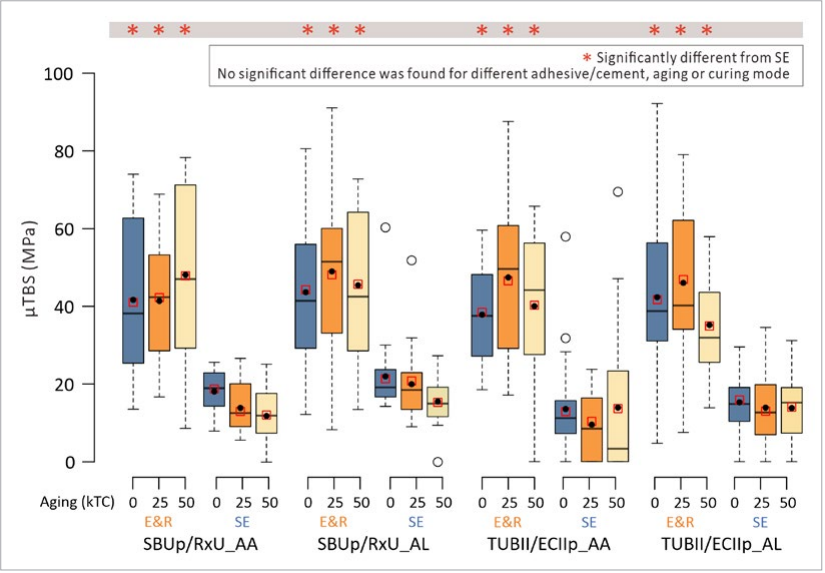
Box-and-whisker plots (Project Part 2) of the 0kTC, 25kTC-aged and 50kTC-aged μTBS of the universal adhesives/ composite cement combinations (UA/CCs) SBUp/RxU and TUBII/ECIIp adhesively luted onto flat dentin, when applied in E&R and SE bonding mode and either following an AA or AL curing mode. The thick horizontal line within each box represents the median μTBS. The black dot within each box represents the mean μTBS. The red square within each box represents the LME fitted mean. The horizontal lines in each box represent, from top to bottom, the maximum μTBS, the upper quartile, the median μTBS, the lower quartile and the minimum μTBS measured for each experimental group (excluding possible outliers). Statistically significant differences in μTBS with the respective reference are indicated using distinct characters.

All μTBS data are numerically detailed in Table 4 and graphically presented as boxplots in Fig 5. As indicated in Table 5, among the four main variables, only the variable ‘bonding mode’ significantly contributed to the statistical model. For the interactions, only the adhesive x bonding mode x aging interaction was significant, meaning the combination of adhesive and bonding mode varied for the three aging periods.

**Table 4:** Microtensile bond strength (µTBS) of the universal adhesive/composite cements (UA/CCs) to flat dentin

µTBS (MPa)*	E&R			SE		
	0kTC	25kTC	50kTC	0kTC	25kTC	50kTC
SBUp/RxU_AA	41.7 ± 19.5 (0/20)	41.4 ± 15.3 (0/20)	48.2 ± 22.7 (0/20)	18.2 ± 5.3 (0/20)	13.9 ± 6.4 (0/20)	11.9 ± 7.4 (3/20)
SBUp/RxU_AL	43.7 ± 18.3 (0/20)	49.0 ± 21.3 (0/20)	45.5 ± 19.3 (0/20)	22.0 ± 10.1 (0/20)	20.0 ± 9.6 (0/20)	15.6 ± 6.0 (1/20)
TUBII/ECIIp_AA	37.8 ± 12.8 (0/20)	47.4 ± 20.1 (0/20)	40.0 ± 18.8 (1/20)	13.5 ± 13.3 (3/20)	9.5 ± 8.9 (7/20)	13.9 ± 19.5 (9/20)
TUBII/ECIIp_AL	42.3 ± 19.9 (0/20)	46.0 ± 18.5 (0/20)	35.2 ± 13.9 (0/20)	15.3 ± 7.3 (1/20)	13.9 ± 10.0 (3/20)	13.8 ± 9.3 (4/20)

*Mean ± SD (ptf/n); SD: standard deviation; ptf: pre-test failure; n: number of µ-specimens.

**Table 5:** Statistical analysis of the fixed factors and interactions of LME model B

	numDF	denDF	F-value	p-value
(Intercept)	1	422	698.1865	<0.0001*
Adhesive	1	36	2.5734	0.1174
Curing	1	36	0.8817	0.3540
Bonding mode	1	422	491.0734	<0.0001*
Aging	2	422	0.9804	0.3760
Adhesive x curing	1	36	0.3810	0.5409
Adhesive x bonding mode	1	422	0.0028	0.9577
Adhesive x aging	2	422	0.4298	0.6509
Curing x bonding mode	1	422	0.9208	0.3378
Curing x aging	2	422	1.5175	0.2204
Bonding mode x aging	2	422	2.9380	0.0541
Adhesive x curing x bonding mode	1	422	0.0039	0.9502
Adhesive x curing x aging	2	422	0.4050	0.6672
Adhesive x bonding mode x aging	2	422	3.6953	0.0256*
Curing x bonding mode x aging	2	422	0.4732	0.6233

*Statistically significant.

The μTBS of SBUp/RxU and TUBII/ECIIp adhesively luted to flat dentin was not significantly different, whether the adhesive was applied in E&R or SE bonding mode, this when tested immediately (0kTC) or upon aging (25kTC and 50kTC). The curing mode did not have a significant impact on the μTBS, considering no significant difference was found for the same UA/CC combinations applied in either AA or AL curing mode. Aging by 25kTC and 50kTC did not significantly decrease the μTBS of any of the UA/CC combinations. Being the only significant difference recorded, UA/CC combinations applied in E&R bonding mode performed significantly better than when they were applied in SE bonding mode, regardless of UA/CC combination.

**Fig 6:**
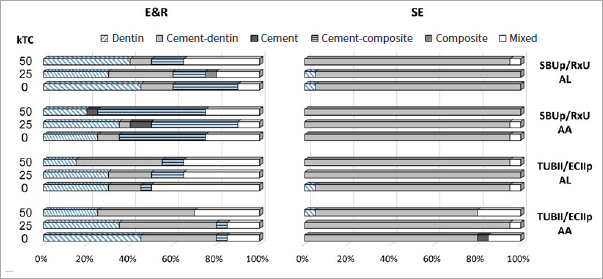
Failure-mode distribution (Project Part 2) of SBUp/RxU and TUBII/ECIIp μ-specimens upon μTBS testing when bonded to flat dentin in E&R and SE bonding mode and AA and AL curing mode. Dentin: cohesive failure in dentin; cement-dentin: adhesive failure at the cement-dentin interface; cement: cohesive failure in cement; cement-composite: adhesive failure at the cement-composite interface; composite: cohesive failure in composite; mixed: mixed failure.

The failure mode distribution examined by stereomicroscopy for the UA/CC combinations adhesively luted to flat dentin is shown in Fig 6. Overall, a balanced failure mode distribution was recorded when the UA/CC combinations were adhesively luted in E&R bonding mode, while adhesive failure at the cement-dentin interface was the predominant failure mode recorded when the UA/CC combinations were applied in SE mode. When applied in E&R bonding mode, TUBII/ECIIp presented more adhesive failures at the cement-dentin interface, while SBUp/RxU revealed more adhesive failures at the cement-composite interface. When applied in SE bonding mode, the failure patterns of SBUp/RxU and TUBII/ECIIp were similar. No distinct difference in failure mode was observed when the UA/CC combinations were adhesively luted in either AL or AA curing mode.

**Fig 7:**
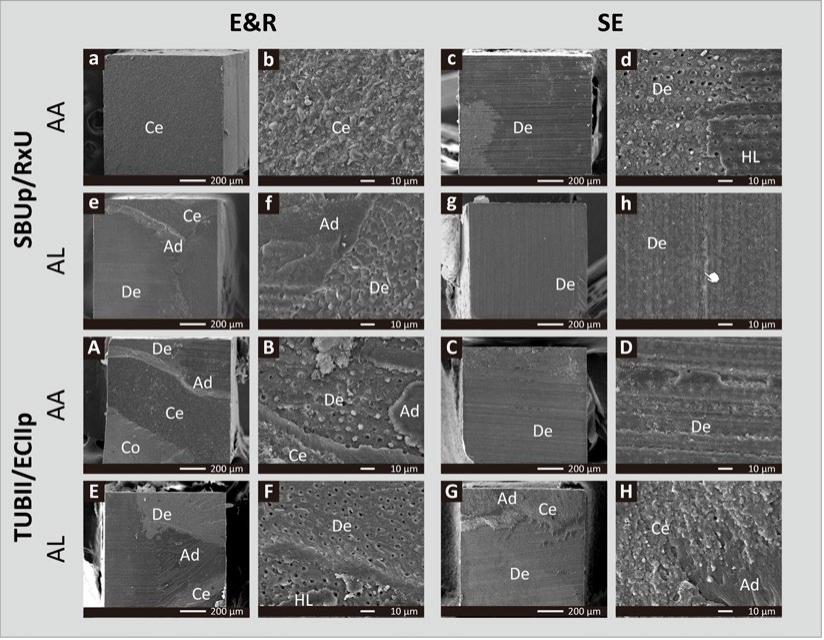
Representative SEM photomicrographs (Project Part 2) of μ-specimens (dentin side) illustrating the failure mode (1) for SBUp/RxU luted in AA curing mode in (a-d) and AL mode in (e-h), and (2) for TUBII/ECIIp when applied in AA curing mode in (A-D) and AL mode in (E-H). (a) SBUp/RxU μ-specimen luted in AA curing mode and E&R bonding mode, revealing a cohesive failure in cement enlarged in (b); (c) SBUp/RxU μ-specimen luted in AA curing mode and SE bonding mode, revealing a primarily adhesive failure at the cement-dentin interface enlarged in (d); (e) SBUp/RxU μ-specimen luted in AL curing mode and E&R bonding mode, revealing a mixed failure enlarged in (f); (g) SBUp/RxU μ-specimen luted in AL curing mode and SE bonding mode, revealing an adhesive failure at the cement-dentin interface enlarged in (h). (A) TUBII/ECIIp μ-specimen applied in AA curing mode and E&R bonding mode, revealing a mixed failure enlarged in (B); (C) TUBII/ECIIp μ-specimen applied in AA curing mode and SE bonding mode, revealing an adhesive failure at the cement-dentin interface enlarged in (D); (E): TUBII/ECIIp μ-specimen applied in AL curing mode and E&R bonding mode, revealing a mixed failure enlarged in (F); (G): TUBII/ECIIp μ-specimen applied in AL curing mode and SE bonding mode, revealing a mixed failure enlarged in (H). Ad: adhesive resin; Ce: cement; Co: composite; De: dentin; HL: hybrid layer.

**Fig 8:**
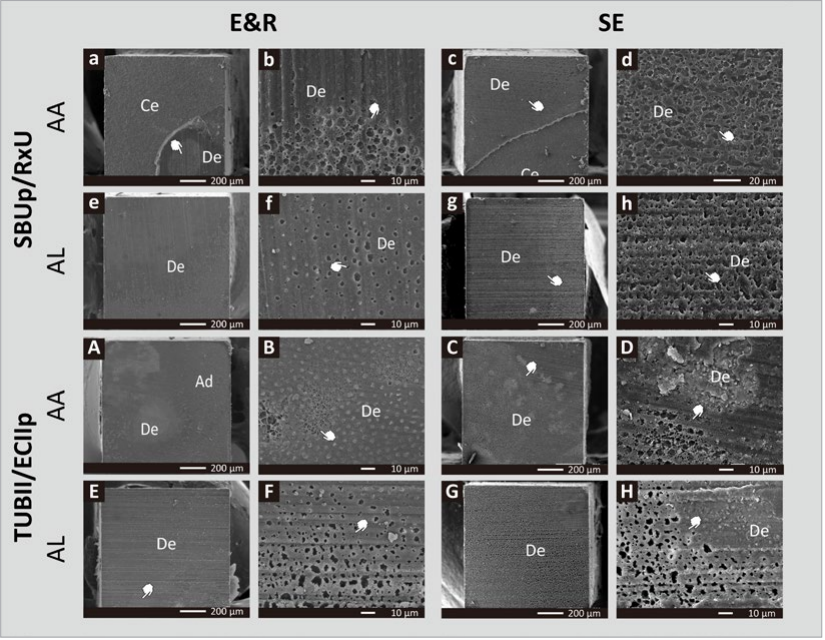
Representative SEM photomicrographs (Project Part 2) showing a large number of irregular interfacial voids (hand pointers) detected at the surface of fractured specimens (composite side) of (1) SBUp/RxU applied in AA curing mode and E&R bonding mode in (a,b) and in SE bonding mode in (c,d), and in AL mode and E&R bonding mode in (e,f) and in SE bonding mode in (g,h), and (2) TUBII/ECIIp applied in AA curing mode and E&R bonding mode in (A,B) and in SE bonding mode in (C,D), and in AL mode and E&R bonding mode in (E,F) and in SE bonding mode in (G,H). Ad: adhesive resin; Ce: cement; De: dentin.

Representative SEM photomicrographs of fractured μ-specimens are shown in Fig 7 (dentin side) and Fig 8 (composite side). Most striking are the interfacial voids observed nearly along the entire fractured surface for UA/CC specimens applied in SE bonding mode, regardless of UA/CC combination and curing mode. When applied in E&R bonding mode, interfacial voids were less intensively observed (Fig 8).

### Adhesive Luting Efficacy of UA/CC Combinations to Lithium-Disilicate Glass-Ceramic (Project Part 3)

**Fig 9:**
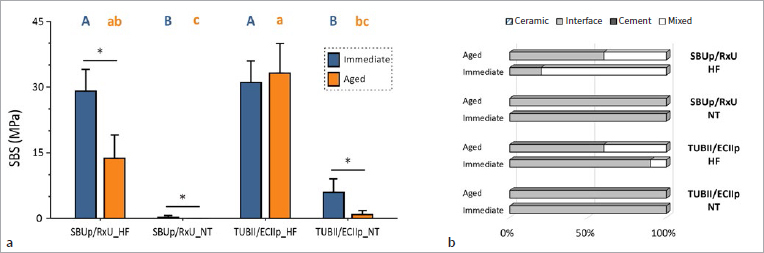
Bar graph (Project Part 3) in (a) presenting the immediate and aged SBS of the universal adhesives/composite cement combinations (UA/CCs) SBUp/RxU and TUBII/ECIIp adhesively luted to lithium-disilicate glass-ceramic (IPS e.max CAD, Ivoclar), with the glass-ceramic surface either etched with hydrofluoric acid (HF) or not (pre-)treated (NT). Groups with the same capital or lowercase letters indicate no significant difference in SBS among the experimental groups when SBS was measured immediately or upon 6-month aging, respectively. Asterisks indicate significant difference between the immediate and aged SBS of the same UA/CC combinations applied following the same procedure. Failure-mode distribution (Project Part 3) in (b) of SBUp/RxU and TUBII/ECIIp specimens upon SBS testing when bonded to glass-ceramic CAD-CAM blocks upon HF etching (HF) or when not (pre-)treated (NT). Ceramic: cohesive failure in glass-ceramic; interface: adhesive failure at the cement-ceramic interface; cement: cohesive failure in cement; mix: mixed failure.

The SBS data are detailed in Table 6 and graphically presented in Fig 9a, with the failure-mode distribution presented in Fig 9b. In general, SBS of the UA/CC combinations with HF treatment was significantly higher than that of the non-etched/treated UA/CCs, whether for SBUp/RxU or TUBII/ECIIp and when measured immediately and upon 6-month aging. No significant difference was found between the UA/CC combinations. Aging upon 6-month water storage significantly decreased SBS for all UA/CC experimental groups except for TUBII/ECIIp_HF. The most predominant failure pattern was adhesive failure at the cement/ceramic interface, in particular when a low SBS was measured.

**Table 6:** Shear bond strength (SBS) of the universal adhesives/composite cements (UA/CCs) to lithium-disilicate glass-ceramic

UA/CC*	SBS (MPa)**	
	Immediate	Aged
SBUp/RxU_HF	29.1 ± 5.0 (0/10)	13.7 ± 5.4 (0/10)
SBUp/RxU_NT	0.3 ± 0.4 (3/10)	0.0 ± 0.0 (10/10)
TUBII/ECIIp_HF	31.1 ± 4.9 (0/10)	33.2 ± 6.8 (0/10)
TUBII/ECIIp_NT	5.9 ± 3.1 (0/10)	0.9 ± 0.9 (2/10)

*HF: hydrofluoric-acid etched; NT: no treatment; **mean ± SD (ptf/n); SD: standard deviation; ptf: pre-test failure; n: number of specimens.

## Discussion

In Project Part 1, the bonding efficacy and interfacial interaction of three UAs (1-step light-curing UA Scotchbond Universal Plus [SBUp, 3M Oral Care], 2-step light-curing UA G2-Bond Universal [G2B, GC], and 1-step self-curing UA Tokuyama Universal Bond II [TUBII, Tokuyama]) were investigated when bonded to flat dentin, in order to determine their best performance when applied in optimal (laboratory) conditions. UAs, like SBUp, were originally designed and developed as simplified one-bottle adhesives that combine the function of the primer and adhesive resin into a single solution with a balanced hydrophilicity/hydrophobicity, to be applied in a simplified 1-step application (2 steps in E&R bonding mode or when selectively etching enamel, a combined E&R-on-enamel/SE-on-dentin bonding mode).^33^ Aiming to better hydrophobically seal the adhesive interface at dentin, 2-step two-bottle UAs, like G2B, were more recently developed and involve the separate application of an acidic hydrophilic primer followed by a solvent-free hydrophobic adhesive resin.^40,47^ Aiming to further simplify the UA application procedure by omitting a separate light-curing step, the self-curing UA TUBII provides a two-bottle UA to be mixed prior to the application without light curing. Besides specific functional monomers, like 4-methacryloxyethyl trimellitic acid (4-MET) for G2B and 3D-SR for TUBII (Table 1), all three UAs investigated in this study contain 10-MDP, to date the most effective functional monomer. Nevertheless, no detailed technical information regarding the 10-MDP concentration (percentage) or purity (quality) is known, although the latter two parameters on their own (besides other factors) have been reported to substantially influence bonding performance.^53,55^

Methacrylate-based dental materials set via free-radical addition polymerization. Dental adhesives are generally light curable,^50^ containing photo-initiators sensitive to blue light in the 400-515 nm range. Although not always listed by the manufacturer, the most commonly used photo-initiator system combines camphorquinone (CQ) with a tertiary amine, as in SBUp. As the most common chemical polymerization initiator, benzoylperoxide is combined with a tertiary amine,^42^ probably as in TUBII, although this information is not unambiguously discernable from the manufacturer’s information (Table 1).

No significant difference in μTBS was detected among the three UAs when they were applied in E&R bonding mode. However, when applied in SE bonding mode, G2B and SBUp significantly outperformed TUBII, for which low μTBS was recorded along with a substantially higher number of ptfs. The first hypothesis, that the bonding efficacy of the self-curing UA would not underperform that of the light-curing adhesives, was hence accepted when applied in E&R bonding mode, but rejected when applied in SE bonding mode. In general, aging did not significantly decrease all of the UAs’ bonding performance (except for G2B applied in SE mode and upon 50k TC). Apart from compositional differences that could result in lower SE bonding effectiveness, the significantly lower bonding effectiveness recorded for TUBII could also be related to its self-curing. Although it is difficult to explain why self-curing would affect the SE bonding mode more than the E&R bonding mode, the much more superficial surface interaction resulting from SE bonding with limited micromechanical interlocking potential (submicron hybrid layer) and even possible smear-layer interference may be a plausible explanation. E&R bonding generates significantly more micromechanical interlocking and completely dissolves/removes all surface smear.

Confirming the lower bonding performance of the self-curing UA TUBII applied in SE bonding mode, SEM fractographic analysis revealed not only substantially more adhesive interfacial failures but also disclosed interfacial voids at the adhesive interface that must have weakened the bond and led to bond failure at lower loading (Fig 3). The occurrence of such interfacial voids should be ascribed to entrapment of water absorbed from the underlying dentin through osmosis.^20,36^ Due to their hydrophilic nature, 1-step adhesives – including 1-step/bottle UAs – act as semi-permeable membranes in an enhanced manner not only prior to polymerization but also even after polymerization, as has been documented previously.^41^ In this study, young third molars (originating from young adolescents) were used; their dentin is highly permeable, which may have promoted interfacial water uptake through osmosis. Such interfacial void production has repeatedly been reported before in the case of adhesive luting, when the adhesive is not separately light cured and solely co-(light) cured with the CC upon seating of the restoration and partial/full cement-excess removal. Delayed curing of the adhesive interface gives water time to be absorbed (see below regarding the Project Part 2 discussion). According to the manufacturer’s instructions, TUBII is advised to be applied within 3 min after mixing the two components, confirming its relatively slow self-curing. Therefore, compared with G2B and SBUp, which were immediately light cured, TUBII was delay-cured, by which the adhesive interface was not immediately stabilized, allowing substantial water droplets to be incorporated at the interface. According to the manufacturer, remaining solvents within TUBII will delay its self-curing, for which reason solvents should be removed as much as possible without over-thinning the adhesive-resin layer. Voids were also observed at the adhesive interface of SBUp with dentin, especially when SBUp was applied in SE bonding mode. This can probably be ascribed to its hydrophilic combined primer/adhesive resin one-bottle design, while much fewer interfacial voids were detected, most likely prevented by immediate light curing. No interfacial voids were observed by TEM, as G2B and SBUp were immediately light cured upon the respective manufacturer’s instructions, while also TUBII must have cured relatively quickly, at least faster when compared to its application in the μTBS study, since a 1-mm-thick adhesive-resin layer of Clearfil SE Bond 2 (Kuraray Noritake) was applied on top of TUBII and immediately light cured for 20 s. The latter may even have resulted in co-curing of TUBII.

Nevertheless, when TUBII was applied in E&R bonding mode, fewer or even no osmosis-induced interfacial voids were observed on the fractured surface of μ-specimens or were only found in a small region. While TUBII applied in E&R bonding mode was also allowed to self-cure with a time delay, favorable bonding effectiveness and bond durability were recorded, comparable to that of the two other light-curing UAs. As very well known and once more confirmed by TEM in Project Part 1b, a thick (3-5 µm) E&R hybrid layer vs a submicron SE hybrid layer was formed, depending on whether the UA was applied following an E&R or an SE bonding mode, respectively. The thicker E&R hybrid layer may have slowed down water absorption from the underlying dentin to reach the adhesive interface, potentially being a plausible reason for the higher μTBS of TUBII recorded when applied in E&R instead of SE bonding mode. It is noteworthy that pulpal pressure in vital teeth can accelerate fluid flow towards the adhesive interface,^30,37^ while it may not have relevant unfavorable interfacial effect in case of relatively impermeable sclerotic/old dentin; hardly any water sorption will occur in non-vital teeth. Further experiments should be conducted to assess whether pulpal pressure can possibly over-wet the dentin surface and impair the adhesion of TUBII even when used in E&R bonding mode.

The lower bond strength of TUBII applied in SE bonding mode, as compared to that of G2B and SBUp, may also be associated with compositional differences between the UAs investigated. All three UAs contain 10-MDP, a highly effective monomer that forms insoluble 10-MDP/Ca salts and therefore is today widespread in recent adhesives, particularly UAs.^12,44^ Previous research reported that 10-MDP forms stable chemical/ionic bonds to HAp, which is important in the wet oral environment to achieve durable adhesion. The functional monomer 10-MDP is more effective than other functional monomers, e.g., 4-MET, as also contained in G2B, and 2-methacryloxyethyl phenyl hydrogen phosphate (phenyl-P).^51^ Compared with SBUp and TUBII, in this study, TEM revealed more prominent nano-layering for G2B, which might be ascribed to different 10-MDP concentrations contained in the respective UAs investigated.^52^ Elaborating further on the speculative explanation that G2B might contain more 10-MDP, SBUp nevertheless did not present significantly lower SE bonding effectiveness, despite an assumed lower concentration of 10-MDP. This may indicate that SBUp was sufficiently optimized for the functional monomer concentration, while TUBII was much less;^19^ again, this is a speculative explanation, as the UA’s detailed composition is not fully revealed.

Self-curing restorative materials were used in dentistry before the introduction of polymer-based dental materials and light curing.^34^ With the development of photo-initiators and more recent, more efficient LED light-curing units, the setting time of light-curing dental materials has dramatically decreased and their curing efficacy has substantially improved. Most resin-based dental materials provide this advantageous ‘on-command’ (light-)curing, except for specific indications when dual-curing provides additional clinical benefits, such as in the case of adhesively luting semi-direct and indirect composite or ceramic (often CAD/CAM) restorations using CC. Indeed, light transmission can be difficult and lead to poorer polymerization when curing must be performed through the restoration, which is highly dependent on the restoration thickness and translucency. It should be noted that even the most opaque dental zirconia ceramics are evolving towards more translucent formulations that better enable light curing through the restorative material.^17,18^ Recent results demonstrate a lack of significantly reduced mechanical properties under such circumstances, for which until recently trade-offs in optical vs mechanical properties would have been necessary.^22,56^

UAs are called universal, as they can also be used to adhesively lute semi-direct/indirect restorations when 1-step UAs are combined with CCs.^27^ Representing the light-curing and self-curing 1-step UA, respectively, the μTBS of SBUp/RxU and TUBII/ECIIp was not found to significantly differ when applied in the same curing mode and bonding mode, either immediately (0kTC) or upon 25kTC- and 50kTC-aging. The bonding efficacy of the two UA/CC combinations, applied either in E&R or SE bonding mode, was comparable irrespective of curing mode. Hence, the second hypothesis that the two UA/CC combinations would not differ in bonding performance, was accepted, as was the third hypothesis, that the curing regimes would not affect the luting efficacy of the CC. This result was in accordance with the findings of previously conducted studies.^2,16^

Combining photo-initiators and chemical initiators, polymerization of dual-curing CCs is initiated first chemically upon mixing of the base and catalysator paste, and second by light upon restoration luting. Reduced light-curing efficacy, due to light attenuation through the restoration, is expected to be compensated by chemical (self-)curing which is initiated sooner but is generally less effective. Light-curing irradiance decreases significantly with increased restoration thickness, up to more than 80% and 95% in 1.5-mm and 3-mm ceramic disks, respectively;^13^ this is obviously highly dependent on the type and translucency of the ceramic. Likewise, light irradiation within root canals, such as when adhesively luting fiber posts, can be inefficient, as light will have difficultly reaching the deeper root areas. Concern has also been raised regarding incompatibility between acidic water-based 1-step adhesives, which includes UAs, and self-curing cements, because the oxygen-inhibition layer of the adhesive may contain sufficient acidic monomers to interact with the amine-based chemical catalytic components of dual-curing CCs and so reduce their polymerization.^8,35^ Insufficient polymerization self-evidently also reduces bond strength.

RxU was bonded to dentin with assistance from the light-curing UA SBUp, while ECIIp was assisted by the self-curing UA TUBII. Nevertheless, SBUp was also not separately light cured as part of the adhesive luting protocol recommended by its manufacturer. SBUp is claimed to be compatible with all dual- and self-curing composite materials because the transition metal-salt added can act as a dual-curing accelerator by catalyzing the decomposition of the peroxide component.^1^

The E&R μTBS of both UA/CC combinations was significantly higher than their SE μTBS. While this was recorded for TUBII when bonded directly to dentin in Project Part 1a, in Project Part 2, this was also documented for SBUp when combined with RxU as part of an adhesive luting protocol. Confirming the lower adhesive luting performance in SE bonding mode, almost 100% adhesive failures at the cement-dentin interface were observed, along with substantially more ptfs, the latter especially upon TC aging and for TUBII/ECIIp. SEM failure analysis provides a clear explanation for the lower SE luting performance, since interfacial voids were detected along almost the entire composite cement-dentin interface, regardless of UA/CC combinations and curing mode. Interfacial voids were more frequently observed when the UA/CC combination was applied in SE than E&R bonding mode. Although previous studies demonstrated that the adhesive should be separately light cured as part of adhesive luting, the adhesives tested were only applied in SE bonding mode.^20,21^ This benefit of separately light curing adhesives is not (or less) obvious when the UA is applied in E&R bonding mode. Further studies should be conducted to verify whether the interfacial void production could be reduced or even eliminated in terms of treating sclerotic impermeable dentin (often the case when luting indirect/semi-direct restorations) or non-vital teeth.

Regarding SBS to lithium-disilicate glass-ceramic, overall, both UA/CC combinations yielded SBS that did not differ significantly, but bonded significantly better to HF-etched than to non-etched glass-ceramic. Hence, the Project Part 3 hypothesis that the adhesive luting efficacy of the UA/CC combinations to lithium-disilicate glass-ceramic would not be affected when the glass-ceramic was not HF etched, was rejected. Although UAs contain acidic functional monomers, such as 10-MDP, they ineffectively (self-)etch glass-ceramic surfaces. Both UAs investigated in this study incorporated γ-MPTES, which has been documented to be more effective than conventional silane coupling agents.^46^ Silane added into one-bottle UAs appeared less effective because silane is insufficiently stable in an aqueous acidic solution.^54^ TUBII separated silane from the acidic functional monomers in a two-bottle configuration, which most likely explains why the SBS of TUBII/ECIIp_HF did not significantly decrease after 6-month water aging in contrast to that recorded for SBUp/RxU_HF.

## Conclusion

For direct bonding, the self-curing 1-step UA performed unsatisfactorily when applied in SE but not in E&R bonding mode, while no difference in bonding effectiveness and durability between SE and E&R bonding was recorded for the light-curing 1-step and 2-step/bottle UAs. When the UA was not separately light cured as part of an adhesive luting protocol, SE did not perform as well as E&R adhesive luting. Adhesively luting glass-ceramic restorations still requires prior HF etching of the glass-ceramic surface, while silane is more effective when separated from the acidic monomer components in UAs.

## Clinical relevance:

When a self-curing UA is employed for direct bonding or the UA is self-cured as part of an adhesive luting protocol, the bonding efficiency of UA applied in E&R bonding mode outperforms that in SE bonding mode.
